# Mendi Mission

**Published:** 1854-04

**Authors:** J. Cutler Tefft

**Affiliations:** Kaw Mendi, West Africa.


### *Mendi Mission.*

MESSRS. JONES, WHITE & MCCURDY—*Gentlemen:*—Perhaps you may have ere this, forgotten me, in the multitudes who call upon you. I was at your office a few evenings before I sailed for my far off mission home, December, 1850, to purchase some gold foil, precip. silver, etc. On informing you of my intended labors among the heathen of benighted Africa, you very generously offered to send to me your excellent "Dental News Letter." I have two objects in addressing you at this time: first, to acknowledge the regular reception of the "Dental News Letter," and to sincerely thank you for it; for although I only practice dentistry now a little for my associates, and occasionally for the natives, yet I love my old profession, and am pleased to hear of the many improvements which are being made in it from year to year. I have been called, when on business at Freetown, the Capitol of Sierra Leone, to practice some for the English, German and Portugeuse, whites settled there as merchants, consuls, ministers, etc., and a few cases for the wealthy native merchants. If there were a few more Europeans setteled at Freetown, it would be a good location for a Dental Surgeon, but as it is, it would not give a living.

While in Sierra Leone, from time to time, I have enjoyed an excellent opportunity to notice the teeth of some twenty different tribes from all parts of Africa—North, South, East, West. They all have better teeth than the whites of America, and those most lately from the slave ships, much the best. Their change of habits, when they come to the colony, many of them becoming servants and cooks in the families of Europeans, where they contract the habit of eating highly seasoned food, and drinking hot tea and coff·e, soon show themselves on their white teeth. As a more certain evidence that it is hot drinks and savory dishes that so seriously affect their teeth, I need only say, that most of the natives for whom I have operated, are those who have been to England for an education. But I think that, perhaps, their excessive use of tobacco may be one of the prominent causes of their soon having bad teeth. Most of them, men and women, smoke very much. Some of the tribes have a very curious custom of filing or cutting either the upper or lower, or both incisors to nearly a point, like the natives of the South Sea Islands. They say they do it to cut 'Yanga,' *i. e.*, to make them look more beautiful. I have never seen any people prize their teeth more highly than the Kroo tribe of Southern Africa. There is no mark of esteem so great to a sweet-heart, as the gift of a tooth. In Sierra Leone, it is an incident of frequent occurrence to meet men of this tribe minus a couple of teeth, *i. e.*, the central superior incisors. A few weeks since, Surgeon St. Clare told me an incident that occurred on board the John Adams, which illustrates my point. One of their Kroomen came to him and requested him to extract two beautiful, sound incisors. The Doctor asked for his reason, and was informed by the honest native, that he wanted to send them back to his own country to his sweet-heart.

One other point and I am done. It occurred to me that I might confer a benefit upon dental or other surgeons, about taking up their abode in a southern clime, to give you my experience in preserving my instrumeuts from rust. When I left America, I had my instruments, as usual, in my dental case, lined with velvet. For about one year, I continued to keep them there, but I was very mnch troubled to keep them in anything like order; indeed, some of them, in spite of my efforts, were much injured. I then packed them in a common tin box, and from that time have had but little trouble with them. From my experience, I think that if surgeons would have a tin box or case made to shut closely with a tray, they could preserve their instruments in damp Southern countries. They should not be packed in any kind of cloth, cotton, etc., but in merely a buckskin, and if oiled at all, let it be with a little mercurial ointment.

Respectfully yours to serve,

J. Cutler Tefft.

*Kaw Mendi, West Africa, July 17th,* 1853.

